# Genetic diversity and population structure of two subspecies of western honey bees (*Apis mellifera* L.) in the Republic of South Africa as revealed by microsatellite genotyping

**DOI:** 10.7717/peerj.8280

**Published:** 2020-01-03

**Authors:** Amin Eimanifar, Johanna T. Pieplow, Alireza Asem, James D. Ellis

**Affiliations:** 1 Entomology and Nematology Department, Honey Bee Research and Extension Laboratory, Gainesville, FL, USA; 2 Independent Senior Scientist, Industrial District, Easton, MD, USA; 3 Molekulare Ökologie, Institut Für Biologie, Molekulare Ökologie, Martin-Luther-Universität Halle-Wittenberg, Halle, Germany; 4 College of Fisheries and Life Science, Hainan Tropical Ocean University, Yucai Road, Sanya, China

**Keywords:** *Apis mellifera scutellate*, *Apis mellifera capensis*, Microsatellite genotyping, Population structure

## Abstract

*Apis mellifera scutellata* and *Apis mellifera capensis*, two native subspecies of western honey bees in the Republic of South Africa (RSA), are important to beekeepers in their native region because beekeepers use these bees for honey production and pollination purposes. Additionally, both bees are important invasive pests outside of their native ranges. Recently, whole mitogenome sequencing and single nucleotide polymorphisms were used to study their genetic diversity. To add to our knowledge of the molecular ecology of both bees, we tested the ability of microsatellites to be used as a tool to discriminate between *A.m. capensis* and *A.m. scutellata*. We analyzed the genetic variability and overall population structure of both bee subspecies and hybrids of the two by genotyping individuals collected from RSA (*N* = 813 bees from 75 apiaries) at 19 microsatellite DNA loci. Overall, populations averaged between 9.2 and 11.3 alleles per locus, with unbiased heterozygosity values ranging from 0.81 to 0.86 per population. Bayesian clustering analyses revealed two distinct evolutionary units, though the results did not match those of earlier morphometric and molecular analyses. This suggests that the microsatellites we tested were not sufficient for subspecies identification purposes, especially for Cape and hybrid bees. Nevertheless, the microsatellite data highlight the considerable genetic diversity within both populations and a larger-than-expected hybridization zone between the natural distributions of *A.m. capensis* and *A.m. scutellata*.

## Introduction

*Apis mellifera* L. (western honey bees) are eusocial bees native to Eurasia and Africa. *A. mellifera* is one of nine honey bee (*Apis* spp.) species, with the other eight species being native to and distributed in Asia (Radloff, Hepburn & Engel, 2011). *A. mellifera* has diversified into approximately 25–30 subspecies which are further classified into six evolutionary groups, or “lineages” (A, M, C, O, Z and Y) (Ruttner, 1988; Garnery, Cornuet & Solignac, 1992; Franck et al., 2000; Alburaki et al., 2013). At least 11 of the *A. mellifera* subspecies are native to Africa. Two subspecies native to the Republic of South Africa (RSA) are of particular interest to us. They are *Apis mellifera scutellata* (the savannah or lowland honey bee) and *Apis mellifera capensis* (the Cape honey bee). These two subspecies hybridize readily in the intermediate areas where their natural distributions overlap (Crewe, Hepburn & Moritz, 1994; Moritz, Beye & Hepburn, 1998; Eimanifar et al., 2018b; Bustamante, Baiser & Ellis, in press).

Both *A.m. scutellata* and *A.m. capensis* are important to beekeepers in the RSA but are considered invasive pests outside of their native ranges (Eimanifar et al., 2018a, 2018b). *A.m. scutellata* is the bee introduced into Brazil in the mid 1950s, later becoming known in the Americas as the “killer bee” (Caron, 2001) due to the heightened defensiveness it exhibits. Cape bees can reproduce thelytokously (worker bees produce diploid, female offspring), a trait that allows them to be social parasites (Aumer et al., 2019). This behavior makes *A.m. capensis* a significant problem in northern parts of the RSA where they are not native. There, they socially parasitize *A.m. scutellata* colonies. Though *A.m. capensis* workers can produce female offspring, they reproduce at much lower rates than do queen honey bees, rendering them unable to support a robust colony population. Consequently, *A.m. scutellata* colonies infested with *A.m. capensis* laying workers suffer dwindling populations and ultimately die. Beekeepers in the RSA call this phenomenon the “capensis calamity” (Allsopp, 1992).

*A.m. capensis* and *A.m. scutellata* show a distinct genetic structure (Jaffe et al., 2009; Neumann et al., 2011; Eimanifar et al., 2018b). Some studies have been published on this topic already, but the sample sizes used render them less informative at the level of subspecies differentiation (Wallberg et al., 2014; Chapman et al., 2015; Harpur et al., 2015; Nelson et al., 2017). Recently, the population structure and genetic diversity of both subspecies was determined using Genotyping-by-Sequencing analyses for 474 individuals, representative of 28 geographical regions in the RSA (Eimanifar et al., 2018b) and whole mitogenome analyses of 25 individuals from across the RSA (Eimanifar et al., 2018a). Eimanifar et al. (2018b) produced a list of 83 divergent SNP loci that can be used to distinguish the two subspecies. In contrast, whole mitogenomes were not useful for distinguishing between the subspecies, though the high genetic diversity of the bees was highlighted (Eimanifar et al., 2018a). In recent years, there have been substantial applications of microsatellite markers to assess the genetic variability, population structure and conservation genetics of many organisms (Wang et al., 2017; Frias-Soler et al., 2018; De Melo Moura et al., 2019; Yu et al., 2019). Thus, to add to our knowledge of the molecular ecology of both bee populations in the RSA, we used 19 microsatellite loci to discern the range-wide genetic variation and population genetic structure of 813 honey bees from 75 different apiaries (29 geographical regions) across the bees’ natural distribution in the RSA. Our microsatellite data, when viewed in context with our earlier work (Eimanifar et al., 2018a, 2018b), highlight the high genetic diversity of the honey bee populations inhabiting the RSA.

## Materials and Methods

### Honey bee sampling and DNA extraction

In April/May 2013 and April 2014, a total of 60+ bees were collected from each of over 1,100 managed honey bee colonies located in 75 different apiaries grouped into 29 geographical regions (1–3 apiaries/region) across the *A.m. scutellata* and *A.m. capensis* distribution in RSA (Mortensen et al., 2016). A region was defined as a cluster of 1–3 apiaries that were located close to one another (i.e., ∼ 5–50 km between apiaries) or was otherwise isolated by itself in instances where a region was composed of only one apiary. The regions were named for the closest city, town, village, or other geographically defined, nearby area. Detailed information on the sampling regions and apiaries, the number of bees analyzed per apiary and the geographical coordinates of the sampling regions are shown in Table 1.

**Table 1:** Summary information for honey bee samples collected in the Republic of South Africa. Apiary No = 75 total apiaries sampled. Sampling regions/apiaries = city/town closest to all apiaries in the region + apiary in/around that city (A–C apiaries). Apiary identifier = code generated from two-letter city/town abbreviation and apiary letter. *N* = number of honey bees examined in a given region. Geographical coordinates = GPS location of each apiary.

Apiary no.	Sampling regions/apiaries	Apiary identifier	*N*	Geographical coordinates
1	Bloemfontein/A	BL/A	14	29.24°S–26.95°E
2	Bloemfontein/B	BL/B	13	29.20°S–27.20°E
3	Bloemfontein/C	BL/C	5	29.24°S–26.94°E
4	Kroonstad/A	KR/A	10	27.58°S–27.30°E
5	Kroonstad/B	KR/B	10	27.33°S–27.50°E
6	Kroonstad/C	KR/C	8	27.27°S–27.50°E
7	Pretoria/A	PT/A	10	25.74°S–28.26°E
8	Pretoria/B	PT/B	17	25.70°S–28.10°E
9	Springbok/A	SP/A	10	29.65°S–17.83°E
10	Springbok/B	SP/B	10	29.71°S–17.78°E
11	Springbok/C	SP/C	10	29.67°S–17.81°E
12	Upington/A	UP/A	10	28.48°S–21.18°E
13	Upington/B	UP/B	10	28.72°S–20.98°E
14	Upington/C	UP/C	9	28.52°S–21.24°E
15	Vryburg/A	VR/A	30	26.96°S–24.76°E
16	Bredasdorp/A	BD/A	10	34.50°S–20.35°E
17	Bredasdorp/B	BD/B	9	34.57°S–20.26°E
18	Bredasdorp/C	BD/C	10	34.53°S–20.29°E
19	Citrusdaal/A	CD/A	9	32.86°S–19.21°E
20	Citrusdaal/B	CD/B	10	32.84°S–19.24°E
21	Citrusdaal/C	CD/C	10	32.67°S–19.06°E
22	Cape Town/A	CT/A	8	33.80°S–18.36°E
23	Cape Town/B	CT/B	12	33.97°S–18.51°E
24	Cape Town/C	CT/C	10	33.96°S–18.45°E
25	George/A	GE/A	8	33.90°S–22.33°E
26	George/B	GE/B	8	33.95°S–22.75°E
27	George/C	GE/C	8	33.98°S–22.47°E
28	Grahamstown/A	GT/A	15	33.31°S–26.49°E
29	Grahamstown/B	GT/B	14	33.37°S–26.42°E
30	Knysna/A	KN/A	15	34.05°S–22.99°E
31	Knysna/B	KN/B	15	34.02°S–22.97°E
32	Langebaan/A	LA/A	10	33.04°S–18.09°E
33	Langebaan/B	LA/B	10	33.00°S–18.31°E
34	Langebaan/C	LA/C	10	33.03°S–18.10°E
35	Laingsburg/A	LB/A	5	33.27°S–20.85°E
36	Laingsburg/B	LB/B	13	33.28°S–20.97°E
37	Laingsburg/C	LB/C	8	33°37′S–21°16′E
38	Moorreesburg/A	MB/A	10	33.10°S–18.74°E
39	Moorreesburg/B	MB/B	10	33.11°S–18.56°E
40	Moorreesburg/C	MB/C	10	33.02°S–18.85°E
41	Modderfontein/A	MF/A	30	33.18°S–25.80°E
42	Oudtshoorn/A	OD/A	10	33.50°S–22.51°E
43	Oudtshoorn/B	OD/B	10	33.53°S–22.54°E
44	Oudtshoorn/C	OD/C	10	33.58°S–22.49°E
45	Plettenburg Bay/A	PB/A	22	34.05°S–23.36°E
46	Plettenburg Bay/B	PB/B	8	34.09°S–23.34°E
47	Port Elizabeth/A	PE/A	10	33.87°S–25.39°E
48	Riversdale/A	RD/A	10	34.31°S–21.50°E
49	Riversdale/B	RD/B	10	34.23°S–21.58°E
50	Riversdale/C	RD/C	10	34.10°S–21.20°E
51	St. Francis/A	SF/A	20	34.17°S–24.81°E
52	Stellenbosch/A	ST/A	10	33.89°S–18.89°E
53	Stellenbosch/B	ST/B	10	33.91°S–18.81°E
54	Stellenbosch/C	ST/C	10	33.85°S–18.82°E
55	Swellendam/A	SW/A	10	34.05°S–20.65°E
56	Swellendam/B	SW/B	10	34.40°S–20.60°E
57	Swellendam/C	SW/C	9	34.19°S–20.30°E
58	Touwsrivier/A	TR/A	8	33.15°S–20.47°E
59	Touwsrivier/B	TR/B	9	33.17°S–20.53°E
60	Touwsrivier/C	TR/C	10	33.17°S–20.26°E
61	Worcester/A	WD/A	10	33.59°S–19.45°E
62	Worcester/B	WD/B	9	33.52°S–19.49°E
63	Worcester/C	WD/C	9	33.62°S–19.69°E
64	Beaufort West/A	BW/A	10	32.34°S–22.64°E
65	Beaufort West/B	BW/B	10	32.34°S–22.64°E
66	Beaufort West/C	BW/C	10	32.34°S–22.62°E
67	East London/A	EL/A	10	33.04°S–27.86°E
68	East London/B	EL/B	8	32.94°S–27.97°E
69	East London/C	EL/C	9	32.97°S–27.90°E
70	Graaff-Reinet/A	GR/A	10	32.25°S–24.53°E
71	Graaff-Reinet/B	GR/B	10	32.17°S–24.56°E
72	Graaff-Reinet/C	GR/C	9	32.26°S–24.54°E
73	Klawer/A	KL/A	10	32.02°S–18.78°E
74	Klawer/B	KL/B	10	32.17°S–18.51°E
75	Klawer/C	KL/C	10	32.10°S–18.84°E
	Total number of bees		813	

The bees were collected and stored by colony in a 50 ml tube containing ≥95% ethanol. The samples were transported (per USDA-APHIS regulations) to the Honey Bee Research and Extension Laboratory at the University of Florida where the identity of each colony (*A.m. capensis, A.m. scutellata* or hybrid) was determined using classical morphometrics conducted on at least ten bees from each colony (for procedure, Bustamante, Baiser & Ellis, in press). The results of the morphometric classification are shown in Table S1. Colony identity was cross-checked using a distribution map published by Hepburn & Radloff (1998). For later analysis, the sample tubes of bees were kept at −80 °C.

For this study, 813 worker bees were sampled from 101 selected colonies across the 75 different apiaries, with 22–33 bees used per region (5–10 bees per colony). The DNA was extracted from the dissected thoraces of each individual bee using Wizard^®^ Genomic DNA Purification kit (Promega, Durham, NC, USA) according to the manufacturer’s instructions. DNA quality was assessed using a 1% agarose gel and quantified using Qubit^®^ 3.0 Fluorometer based on manufacturer’s guidelines (Thermo Scientific Inc., Waltham, MA, USA).

### Microsatellite genotyping

Nineteen microsatellite loci were selected from previous studies and deemed appropriate candidates for the genotyping of the target bee populations (Shaibi, Lattorff & Moritz, 2008; Chen et al., 2005; Alburaki et al., 2013; Rowe et al., 1997; Solignac et al., 2003; Weinstock et al., 2006). The forward primers were labeled at the 5′ end with one of four fluorescent dyes (FAM, VIC, PET and NED; Applied Biosystems, Foster, CA, USA) and distributed into six multiplex PCR reactions. A list of labeled primers and associated information is included in Table S2. PCR reactions were performed in a thermal cycler (Eppendorf, Hamburg, Germany) in 20 µl volumes containing 10 µl of Master Mix Maxima Hot Start 2X Qiagen, 0.6 µl of each primer (10 pmol/µl) and one µl of DNA (>10 ng/µl). The PCR program started with a denaturation phase at 95 °C for 5 min, followed by 35 cycles of 30 s at 95 °C, 30 s at 55 °C, 30 s at 72 °C and a final elongation phase at 72 °C for 30 min. The resulting PCR products were diluted 25–100 fold before genotyping and submitted to the University of Florida’s Interdisciplinary Center for Biotechnology Research for sequencing. A volume of 990 µl Hi-Di™ Formamide (Catalog # 4311320; Life Technologies, Grand Island, NY, USA) was mixed with 10 µl of the GeneScan™ 600 LIZ size standard (Catalog # 1406056; Life Technologies, Grand Island, NY, USA). A volume of 10 µl of the solution was mixed with one µl of PCR product per sample and distributed in a 96 well plate. Fragment lengths were determined using a 3730*1 DNA Analyzer (Model 325-0020; Applied Biosystems, Foster City, CA, USA) and alleles were scored using GeneMarker version 2.4.0 (Applied Biosystems, Foster City, CA, USA) (Hulce et al., 2011). All electropherograms were checked manually and confirmed for further statistical analyses.

### Statistical analyses

#### Population genetic analyses

MICRO-CHECKER 2.2.3 was used to identify the likelihood of scoring errors due to null alleles, stutter bands and large allele dropout (Van Oosterhout et al., 2004). We genotyped 10% of the samples twice to evaluate the rate of genotyping errors. Microsatellite diversity was evaluated by calculating the mean number of alleles per locus (*Na*), the number of effective alleles (*Ne*), observed (*H*_obs_) and expected (*H*_exp_) heterozygosities, unbiased expected heterozygosity (u*He*), Shannon index (*I*) per sampling region, and the number of private alleles per region (NP) using GenAlEx 6.5 (Peakall & Smouse, 2012). The inbreeding coefficient (*F*_IS_) was estimated based on Wrights *F*-statistics using GenAlEx 6.5 (Peakall & Smouse, 2012). Allelic richness (Ar) per sampling region was calculated with FSTAT 2.9.3 (Goudet, 2001) using the rarefaction method. Bee samples from BW, CD, PE, and WD were not included in the population diversity analyses because the microsatellites assigned them to bee groups that were different from those to which they were assigned using single nucleotide polymorphisms (SNPs) (Eimanifar et al., 2018b) and morphometrics (Bustamante, Baiser & Ellis, in press). Thus, their identities were questionable, making their assignment to a bee group for population analysis injudicious.

The deviation of genetic markers from Hardy–Weinberg equilibrium was examined for each combination of locus and region based on the exact test following Markov chain parameters including dememorization = 5,000, batches = 5,000, iterations per batch = 1,000 (Guo & Thompson, 1992) as implemented in Genepop 4.2 (Raymond & Rousset, 1995). Overall population differentiation indices were calculated among regions (again, excluding bees from BW, CD, PE and WD) based on Wrights *F*-statistics index (*F*_ST_) using GenAlEx 6.5 (Peakall & Smouse, 2012). We also calculated Hedrick’s G’_ST_ and Jost’s D for each subspecies as genetic differentiation indicated by *F*_ST_ may remain undetected for highly variable, multi-allelic markers such as microsatellites (Meirmans & Hedrick, 2011). G’_ST_ is the original G_ST_ as defined by Nei (1978) and standardized by the maximum value it can obtain (G_ST (max)_) (Hedrick, 2005). Jost’s D is calculated based on the effective number of alleles instead of heterozygosity, which is considered a more intuitive diversity estimate (Jost, 2008). All three pairwise genetic differentiation metrics were calculated and their significance was inferred based on 9,999 permutations in GenAlEx 6.5 (Peakall & Smouse, 2012). The overall genetic differentiation indices (*F*_ST_, G’_ST_ and Jost’s D) were compared statistically between subspecies using a one-way ANOVA test as implemented in SPSS Statistica (IBM Corp, 2013). A significance value of 95% for confidence intervals was applied to analyze the pairwise data set. The matrix of genetic distance was calculated based on Nei index among regions using GenAlEx 6.5 (Peakall & Smouse, 2012).

Partitioning of genetic variation between and within regions was determined by a hierarchical analysis of molecular variance in Arlequin v. 3.5 package (Excoffier & Lischer, 2010). The significance of these values was examined based on 9,999 permutations. The structure of defining regions was adjusted based on clustering patterns of honey bees distributions described by Hepburn & Radloff (1998).

#### Bayesian population structure analysis

Analyses of genetic clustering among regions were performed using STRUCTURE 2.3.4 (Pritchard, Stephens & Donnelly, 2000). An admixture model with correlated allele frequencies were assumed. We applied a number of clusters (*K*), varying from 1 to 10, with five independent runs per each *K* to estimate the most reliable number of genetic clusters (*K*) using the posterior probability (LnP (N/K)) (Falush, Stephens & Pritchard, 2007) and ad hoc quantity *DK* for each *K* partition. We conducted the analysis with no prior information about population identity with the following parameters: 100,000 pre-burn steps and 750,000 post-burn iterations of the MCMC algorithm for each run. Posterior probability changes with respect to *K* between different runs were assigned as a method for determining the true *K* value (Evanno, Regnaut & Goudet, 2005). The most likely value for *K* was estimated applying Evanno’s ∆*K* method (Evanno, Regnaut & Goudet, 2005) using STRUCTURE HARVESTER (Earl & Von Holdt, 2012). We used individual Q matrices to visualize structure bar plots using STRUCTURE PLOT (Ramasamy et al., 2014).

## Results

The result from MICRO-CHECKER revealed no issues with scoring alleles due to stutters or allelic dropout in any of the 19 loci. We detected the occurrence of homozygote excess for three loci (UN351, HB-SEX-01 and HB-THE-03), likely indicating the occurrence of null alleles. Because of this, all datasets were rerun excluding these loci. In each case, the results were similar to those obtained using all 19 loci, so we included all loci in the analyses. In order to estimate population genetic indices, we divided all regions based on subspecies identity determined by Eimanifar et al. (2018b) and Bustamante, Baiser & Ellis (in press). The results of the population genetics indices are depicted in Table 2 for each region.

**Table 2:** Population genetic characteristics, determined using 19 microsatellite loci of honey bees sampled from 25 geographical regions in the Republic of South Africa (excluding bees from BW, CD, WD and PE—see text). Region abbreviations are explained in Table 1. Bee identity was determined using Eimanifar et al. (2018a, 2018b) and (Bustamante, Baiser & Ellis, in press). Abbreviations: *N*_a_, mean number of observed allele per locus; *N*_e_, mean number of effective population size; *Ar*, mean allelic richness; *H*_obs_, observed heterozygosity; *H*_exp_, expected heterozygosity; u*He*, mean unbiased estimate of expected heterozygosity; *I*, Shannon index; *F*_IS_; Fixation index and *N*_P_ (%), percentage of mean number of private alleles per region. The standard deviation is indicated in parentheses. The data are the mean with s.e. below in parentheses (Mean (s.e.)).

Pop.	*Na*	*Ne*	*Ar*	*H*_obs_	*H*_exp_	u *He*	*I*	*F*_IS_	*N*p (%)
*A.m. scutellata* regions									
BL	10.7 (0.8)	6.4 (0.5)	8.73 (2.33)	0.79 (0.04)	0.83 (0.01)	0.84 (0.01)	2 (0.08)	0.05 (0.04)	15.8 (8.6)
KR	10.4 (0.7)	6.5 (0.5)	8.83 (2.27)	0.75 (0.03)	0.83 (0.01)	0.85 (0.01)	2.01 (0.07)	0.10 (0.03)	0 (0)
PT	10.7 (0.7)	5.9 (0.4)	8.86 (2.18)	0.79 (0.03)	0.82 (0.01)	0.83 (0.01)	1.97 (0.07)	0.03 (0.03)	26.3 (10.4)
SP	9.9 (0.5)	5.7 (0.3)	8.14 (1.51)	0.77 (0.04)	0.81 (0.01)	0.83 (0.01)	1.92 (0.05)	0.06 (0.04)	10.5 (7.2)
UP	9.8 (0.6)	5.8 (0.4)	8.19 (1.92)	0.74 (0.03)	0.81 (0.01)	0.83 (0.01)	1.91 (0.07)	0.09 (0.03)	5.3 (5.3)
VR	9.9 (0.6)	5.4 (0.3)	8.00 (1.65)	0.74 (0.04)	0.80 (0.01)	0.82 (0.01)	1.88 (0.06)	0.08 (0.04)	10.5 (7.2)
*A.m. scutellata* mean (SE)	10.23 (0.42)	5.95 (0.42)	8.46 (0.39)	0.76 (0.02)	0.82 (0.01)	0.83 (0.01)	1.95 (0.05)	0.07 (0.03)	11.4 (9.06)
*A.m. capensis* regions									
BD	9.8 (0.6)	5.8 (0.4)	8.11 (1.81)	0.78 (0.04)	0.81 (0.02)	0.82 (0.02)	1.91 (0.07)	0.04 (0.04)	0 (0)
CT	9.3 (0.5)	5.3 (0.3)	7.84 (1.50)	0.73 (0.05)	0.80 (0.01)	0.81 (0.01)	1.86 (0.06)	0.09 (0.05)	0 (0)
GE	9.2 (0.5)	5.3 (0.3)	8.13 (1.65)	0.74 (0.03)	0.80 (0.01)	0.81 (0.01)	1.86 (0.06)	0.07 (0.04)	15.8 (8.6)
LB	10.3 (0.6)	6.4 (0.4)	8.91 (2.15)	0.75 (0.04)	0.83 (0.01)	0.85 (0.01)	2.01 (0.07)	0.10 (0.04)	0 (0)
LA	9.6 (0.5)	5.4 (0.4)	7.92 (1.53)	0.77 (0.03)	0.80 (0.01)	0.82 (0.01)	1.87 (0.06)	0.04 (0.04)	0 (0)
MB	10.2 (0.6)	6.1 (0.5)	8.55 (1.90)	0.77 (0.03)	0.81 (0.02)	0.83 (0.02)	1.96 (0.08)	0.06 (0.03)	10.5 (7.2)
OD	11.3 (0.6)	6.3 (0.4)	9.30 (1.91)	0.73 (0.04)	0.83 (0.01)	0.84 (0.01)	2.05 (0.06)	0.12 (0.05)	0 (0)
RD	10.5 (0.7)	6.1 (0.4)	8.58 (1.95)	0.80 (0.04)	0.82 (0.02)	0.84 (0.02)	1.98 (0.07)	0.03 (0.03)	10.5 (7.2)
ST	9.7 (0.5)	5.5 (0.3)	8.08 (1.49)	0.76 (0.04)	0.81 (0.01)	0.82 (0.01)	1.89 (0.05)	0.06 (0.04)	5.3 (5.3)
SW	9.6 (0.7)	5.9 (0.5)	8.21 (2.17)	0.70 (0.04)	0.81 (0.02)	0.83 (0.02)	1.92 (0.07)	0.14 (0.05)	5.3 (5.3)
TR	9.8 (0.6)	6.1 (0.4)	8.50 (2.10)	0.71 (0.05)	0.82 (0.01)	0.84 (0.01)	1.94 (0.07)	0.13 (0.05)	5.3 (5.3)
*A.m. capensis* mean (SE)	9.94 (0.60)	5.84 (0.40)	8.38 (0.44)	0.75 (0.03)	0.81 (0.01)	0.83 (0.01)	1.93 (0.06)	0.08 (0.04)	4.79 (5.49)
Hybrid regions									
EL	10.8 (0.5)	6.2 (0.4)	9.00 (1.67)	0.73 (0.03)	0.82 (0.01)	0.84 (0.02)	2 (0.06)	0.12 (0.04)	15.8 (8.6)
GR	9.5 (0.6)	5.7 (0.4)	7.92 (1.83)	0.77 (0.03)	0.81 (0.02)	0.82 (0.02)	1.88 (0.07)	0.05 (0.03)	0 (0)
GT	10.1 (0.5)	5.9 (0.3)	8.44 (1.50)	0.75 (0.04)	0.82 (0.01)	0.84 (0.01)	1.96 (0.05)	0.09 (0.05)	10.5 (7.2)
KL	10.8 (0.6)	6.9 (0.4)	9.10 (1.86)	0.75 (0.04)	0.84 (0.01)	0.86 (0.01)	2.07 (0.06)	0.11 (0.04)	15.8 (8.6)
KN	9.9 (0.5)	6.1 (0.4)	8.36 (1.63)	0.73 (0.03)	0.82 (0.01)	0.84 (0.01)	1.96 (0.06)	0.11 (0.04)	5.3 (5.3)
MF	10.0 (0.7)	5.9 (0.3)	8.35 (1.77)	0.77 (0.03)	0.82 (0.02)	0.83 (0.02)	1.94 (0.06)	0.06 (0.03)	21.1 (9.6)
PB	10.3 (0.5)	5.7 (0.3)	8.28 (1.60)	0.71 (0.03)	0.81 (0.01)	0.83 (0.01)	1.92 (0.06)	0.12 (0.04)	15.8 (8.6)
SF	8.3 (0.5)	5.3 (0.3)	7.59 (1.63)	0.75 (0.05)	0.80 (0.01)	0.82 (0.01)	1.80 (0.05)	0.06 (0.06)	5.3 (5.3)
Hybrid mean (SE)	9.96 (0.80)	5.96 (0.47)	8.38 (0.50)	0.75 (0.02)	0.82 (0.01)	0.84 (0.01)	1.94 (0.08)	0.09 (0.03)	11.20 (7.14)

**Figure 1:**
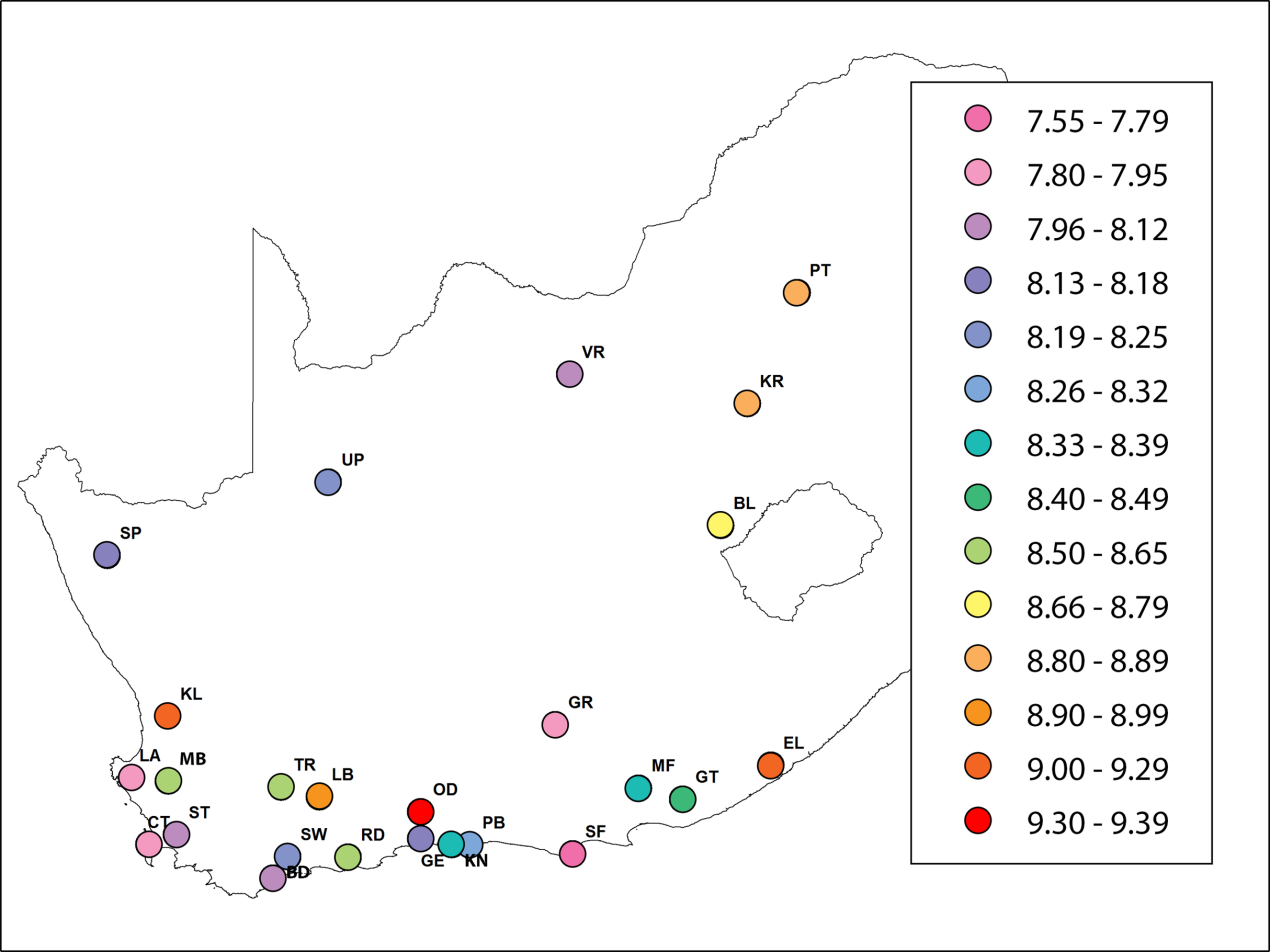
A heat map showing the value of allelic richness of honey bees from 29 regions in the Republic of South Africa (generated using ArcGIS). The abbreviations of the regions are explained in Table 1.

The mean number of alleles per locus was 10.01 ± 0.11 (mean ± s.e. here and hereafter) and varied from 5.8 (A24) to 13.44 (A107). The mean number of alleles for *A.m. scutellata* (six regions), *A.m. capensis* (11 regions) and hybrid populations (eight regions) was 10.23 ± 0.42, 9.94 ± 0.6 and 9.96 ± 0.8, respectively. The *H*_exp_ value ranged from 0.72 at locus A24 to 0.87 at locus A14, while the *H*_obs_ value ranged from 0.35 at locus HB-SEX-01 to 0.88 at locus AC6. The *H*_exp_ value across all loci was highest in the KL region, with an average of 0.84. The lowest *H*_exp_ values were found in the VR, CT, GE, LA and SF regions, with an average of 0.8, respectively. Observed heterozygosity ranged from 0.7 in the SW region to 0.8 in the RD region. Mean *H*_exp_ and *H*_obs_ values were 0.81 and 0.75, respectively. The highest and lowest unbiased expected heterozygosities were found in KL (0.86), and CT/GE (0.81), respectively. Multilocus values of *F*_IS_ per region ranged from 0.03 (PT and RD regions) to 0.14 (SW region), and the overall value was significantly positive 0.08 ± 0.008. Allelic richness among regions of *A.m. scutellata* and *capensis* varied from 7.84 (CT region) to 9.3 (OD region). In hybrid regions, it varied from 7.59 (SF region) to 9.1 (KL region) (Table 2; Fig. 1).

Hardy–Weinberg equilibrium tests were performed for each region and each locus. No regions or loci deviated from Hardy–Weinberg equilibrium (*P* < 0.05). The percentage of the mean number of private alleles varied from 0 (KR, BD, CT, LB, LA, OD, and GR regions) to 26.3% (PT region). The percent mean number of private alleles in *A.m. scutellata*, *A.m. capensis*, and hybrid bees was 11.4%, 4.79% and 11.2% respectively (Table 2). The highest and the lowest value of genetic distance was observed between UP-RD and MF-ST (0.68), and BD-RD (0.30), respectively (Table S3). *A.m. capensis* had higher *F*_ST_, G’_ST_ and Jost’s D values than did *A.m. scutellata* and hybrid bees but there was no significant difference observed between them (Table 3).

**Table 3:** Estimation of pairwise mean genetic differentiation indices for *A.m. scutellata*, *A.m. capensis* and hybrid honey bees collected from the Republic of South Africa. Columnar means sharing the same letter are not significantly different; ANOVA, Tukey test, *P* > 0.05. *F* value refers to the ratio of the between groups to within groups mean square. All tests are calculated based on 9,999 bootstraps. CI: 95% confidence intervals.

	*F*_ST_ (mean ± SD) [95% CI]	G’_ST_ (mean ± SD) [95% CI]	Jost’s D (mean ± SD) [95% CI]
*A.m. scutellata*	0.06 ± 0.01^a^ [0.04–0.11]	0.04 ± 0.01^a^ [0.02–0.08]	0.29 ± 0.09^a^ [0.12–0.44]
*A.m. capensis*	0.07 ± 0.01^a^ [0.05–0.12]	0.05 ± 0.01^a^ [0.03–0.09]	0.31 ± 0.10^a^ [0.18–0.53]
Hybrid	0.06 ± 0.01^a^ [0.05–0.09]	0.04 ± 0.01^a^ [0.03–0.07]	0.28 ± 0.08^a^ [0.13–0.50]
*F*-value	2.41	2.28	0.498
*P* value	0.099	0.111	0.610

**Figure 2:**
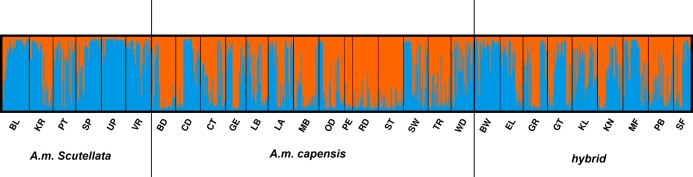
Bayesian clustering assignment of 813 honey bees using STRUCTURE. Bayesian clustering assignment of 813 honey bees of *A.m. scutellata*, *A.m. capensis* and hybrid colonies from 29 geographical regions in the Republic of South Africa using STRUCTURE. The regions are defined in Table 1. Cluster analysis of subspecies are shown at *K* = 2, without prior information of population identity. The different regions are depicted on the *X*-axis and were defined using SNPs (Eimanifar et al., 2018b). Each honey bee is represented by a bar that is, segmented into two colors based on the assignment into inferred clusters given the assumption of *K* populations. The length of the colored segment is the estimated proportion of alleles of the individuals belonging to that cluster. The analysis was performed in five replicates for each *K* so the replicate with the highest likelihood is shown.

**Figure 3:**
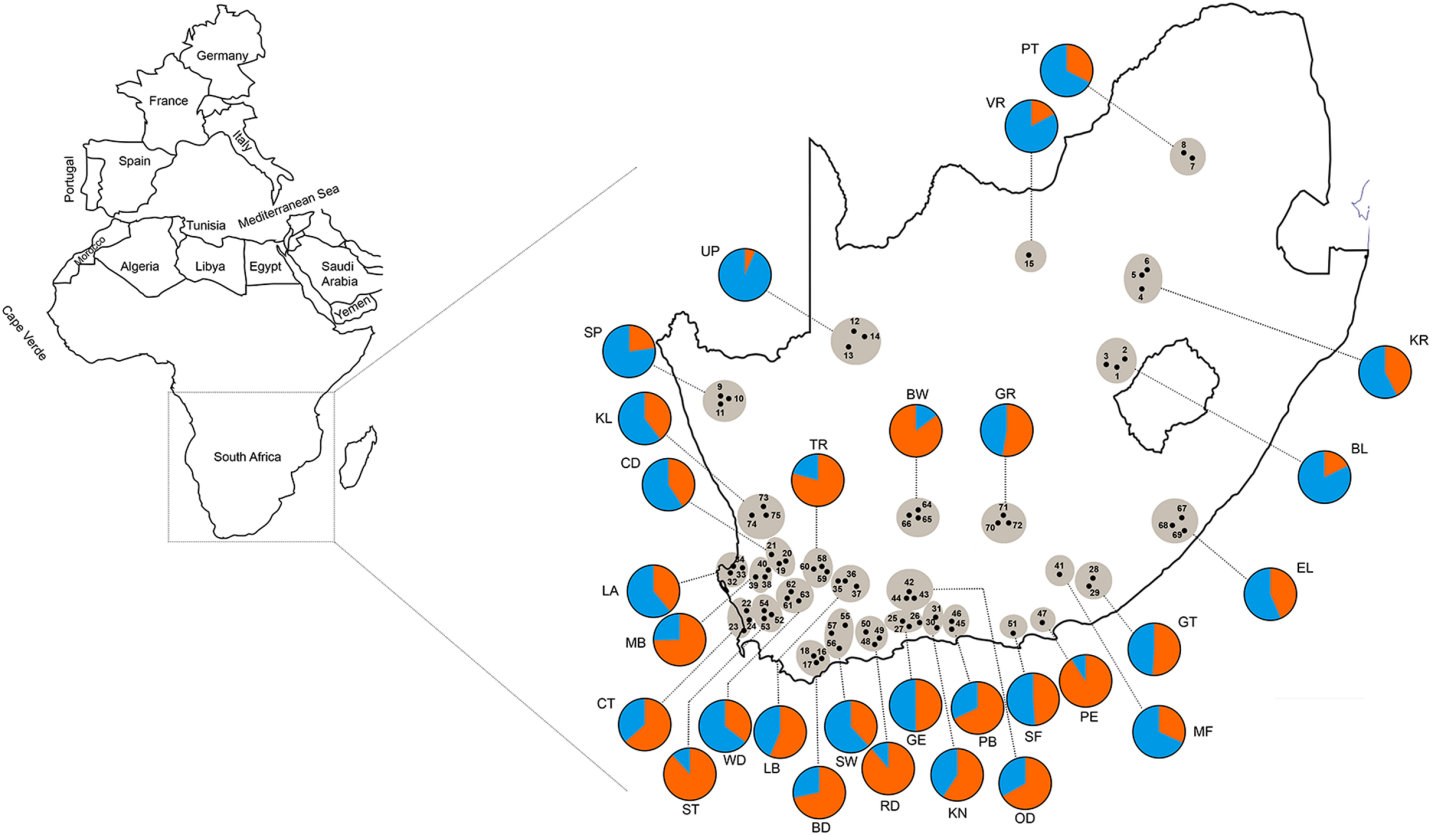
Geographic distribution of 75 sampling apiaries (black dots) of honey bees in the Republic of South Africa. Adjacent apiaries around each town are clustered into a single region (29 total regions grayed in the figure) abbreviated with the name of the closest town (the region abbreviations are defined in Table 1). The pie charts represent the composition of the two genetic clusters for each geographical region shown as blue (more *A.m. scutellata*) and orange (more *A.m. capensis*). The colors indicate the different proportion of allele frequencies assigned to bees from each region.

The AMOVA results indicated that most of the genetic variation (78.97%) was attributed within populations (i.e., between bees within a region) with only 5.05% of the variation occurring between the 25 regional populations (Table 4). In the STRUCTURE analysis, the most likely number of clusters across regions was estimated based on likelihood and Delta *K* scores across regions. Two genetically heterogeneous clusters were detected by STRUCUTRE as the value of Delta *K* was greatest at *K* = 2. The average value of posterior probabilities and Delta *K* for each *K* are shown in Table 5. The results suggest that the sampled honey bees belong to two large genetic groups at the subspecies level, with evidence of admixture patterns across the regions of the two defined populations (Fig. 2). The regions were assigned with unequal probability to each genetic group (Fig. 3). When assigning all genotypes at the subspecies level for morphometrically *A.m. scutellata*-like bees, 23% and 77% of the bees were assigned to the K1 and K2 genetic groups, respectively. For morphometrically *A.m. capensis*-like bees, 63% and 37% of the bees were assigned to the K1 and K2 genetic groups respectively. For morphometrically hybrid bees, 45.5% and 54.5% were assigned to the K1 and K2 genetic groups respective (Fig. 3). The microsatellite genotypes generated for all honey bee’s analyzed are provided in Table S4.

**Table 4:** Analysis of molecular variance on genetic partitioning for honey bees. The results of an Analysis of Molecular Variance on genetic partitioning for honey bees from the Republic of South Africa using 19 microsatellite loci and recognizing three populations (*A.m. scutellata*, *A.m. capensis* and hybrid bees).

Source of variation	df	Sum of squares	Estimated variance	Percentage of variation (%)
Between regional populations	24	812.89	0.42	5.05
Among individuals	691	6,491.30	1.35	15.98
Within populations (between bees within a region)	716	4,788.50	6.68	78.97
Total	1,431	12,092.69	8.56	100

**Table 5:** Estimated posterior probabilities and Delta *K* for each K partition.

K	Reps	Mean LnP (K)	Stdev LnP (K)	Ln′ (K)	*|Ln*″(K)*|*	Delta *K*
1	5	−65,504.2	0.49295	–	–	–
2	5	−64,668.3	2.65	835.90	248.80	93.85
3	5	−64,081.2	18.37	587.10	205.68	11.20
4	5	−63,699.8	308.14	381.42	262.48	0.85
5	5	−63,055.9	19.78	643.90	179.82	9.09
6	5	−62,591.8	43.82	464.08	26.76	0.61
7	5	−62,154.5	97.66	437.32	48.46	0.50
8	5	−61,765.7	190.04	388.86	62.70	0.33
9	5	−61,314.1	52.00	451.56	244.74	4.71
10	5	−61,107.3	279.83	206.82	–	–

## Discussion

In the present study, we present information resulting from microsatellite genotyping to evaluate the detailed genetic diversity and population structure of two subspecies (*A.m. scutellata* and *A.m. capensis*) and their hybrids from 29 regions in the RSA. Microsatellite loci have been widely used to characterize the structure of honey bee populations across different geographical regions since they have a high degree of sensitivity and provide a reasonable level of polymorphism (Franck et al., 2001; Loucif-Ayad et al., 2015; Techer et al., 2015).

### Genetic diversity

In an analysis of 19 microsatellite loci, we detected a high level of genetic variability based on the mean number of alleles observed per microsatellite locus and heterozygosity within and among examined honey bee regions. The high level of genetic diversity observed for all honey bee groups was consistent with that shown in previous studies (Estoup et al., 1995; Franck et al., 1998; Jaffe et al., 2010; Eimanifar et al., 2018a, 2018b). This may be true in RSA given the high densities of wild honey bee colonies in certain areas of the country (Vaudo et al., 2012a, 2012b).

The microsatellites we used tended to classify bees from certain regions as hybrids, more so than did SNPs, which tended to separate the subspecies more predictably (Eimanifar et al., 2018b). In fact, both the *A.m. capensis* and *A.m. scutellata* regions in RSA were smaller when defined by microsatellites than they were when defined using SNPs or whole mitogenomes (Eimanifar et al., 2018a, 2018b). Consequently, we feel the microsatellites possibly overestimated the size of the hybrid zone. In contrast, it is possible that the microsatellites provided evidence of greater hybridization between the two subspecies than first thought.

As expected, honey bees in the hybrid region in RSA contain a gene pool derived from hybridization of *A.m. scutellata* and *A.m. capensis* bees. The hybrid bees in our study had many polymorphic alleles that likely originated from recombination of alleles of the two subspecies (Alda & Doadrio, 2014). We suspect that the occurrence of new alleles in the hybrid bees could be a result of hybridization of the two subspecies, as is known to happen for populations in hybrid zones in general (Arnold et al., 1999).

The mean number of alleles observed across all loci (*Na* = 10.01) and the mean value of observed heterozygosity (*H*_obs_ = 0.75) were greater for bee populations in our study (*N* = 716) than those reported in the literature for Algeria (*N* = 414, *Na* = 9.5, *H*_obs_ = 0.69) (Loucif-Ayad et al., 2015), Croatia (*N* = 225, *Na* = 9.25, *H*_obs_ = 0.67) (Muñoz et al., 2009) and on Rodrigues Island (*N* = 524, *Na* = 7.63, *H*_obs_ = 0.64) (Techer et al., 2016). The greater mean number of alleles/locus in our study than in others likely resulted from our large sample size and generally large populations of the two subspecies we examined (Loucif-Ayad et al., 2015). African honey bees exhibit several exceptional characteristics, including pronounced migratory behavior (absconding and swarming), which possibly could explain their high level of genetic diversity with low levels of differentiation (Franck et al., 1998; Hepburn & Radloff, 1998).

### Population genetic structure

The set of microsatellite markers we used yielded a distinctive population structure within the examined groups, though the microsatellite results did not align completely with those of the morphometric (Bustamante, Baiser & Ellis, in press) and other molecular results (Eimanifar et al., 2018b). Nevertheless, our STRUCTURE results support the presence of some genetic structure at the subspecies level, reinforcing other work showing that there are two different gene pools existing in *A.m. capensis* and *A.m. scutellata* honey bees from the RSA (Eimanifar et al., 2018a, 2018b).

We found additional evidence of the allelic introgression of *A.m. capensis* from their native range into that of *A.m. scutellata* regions (Neumann & Moritz, 2002; Moritz et al., 2008). These results highlight the decades-old, continuing impact of the “*capensis* calamity” on *A.m. scutellata* colonies in the RSA. Our STRUCUTRE results (Fig. 3) suggest that the *A.m. capensis*/*scutellata* hybrid zone has moved north and east from that originally delineated by Hepburn & Radloff (1998) over two decades ago.

In conclusion, honey bees in the RSA are composed of two genetically different populations, both with significant genetic structure. Given our large sample size and analytical tools, we show two distinct evolutionary units, though the results did not match those of earlier morphometric and molecular analyses (Eimanifar et al., 2018b; Bustamante, Baiser & Ellis, in press), suggesting that the microsatellites we tested were not sufficient for subspecies identification purposes, especially for Cape and hybrid bees. Nevertheless, our data highlight the considerable genetic diversity within both populations, a possibly larger-than-expected hybridization zone between the natural distributions of *A.m. capensis* and *A.m. scutellata*, and evidence of the expansion of *A.m. capensis* into *A.m. scutellata* regions. The genetic introgression of *A.m. capensis* into *A.m. scutellata* territory continues from the days of the “*capensis* calamity” and highlights the invasive potential of *A.m. capensis*. Our data suggest that the hybrid zone has expanded in the RSA beyond that shown using morphological data (Hepburn & Radloff, 1998; Bustamante, Baiser & Ellis, in press), possibly owing to migratory beekeeping activities in the RSA.

## Supplemental Information

10.7717/peerj.8280/supp-1 Supplemental Information 1 Summary information for honey bee samples collected in the Republic of South Africa.

10.7717/peerj.8280/supp-2 Supplemental Information 2 The multiplex groups of microsatellite loci.

10.7717/peerj.8280/supp-3 Supplemental Information 3 The pairwise of Nei’s (1978) genetic distances among 25 geographical regions.

10.7717/peerj.8280/supp-4 Supplemental Information 4 Raw microsatellite data.

## Additional Information and Declarations

### Field Study Permissions

The following information was supplied relating to field study approvals (i.e., approving body and any reference numbers): Field permits were not required. Samples were collected from beekeeper colonies, not wild colonies, with the beekeepers’ permission.

### Data Availability

The following information was supplied regarding data availability: Raw data is available in Table S4.
