# Supplementary material for: Genetic diversity and population structure of two subspecies of western honey bees (Apis mellifera L.) in the Republic of South Africa as revealed by microsatellite genotyping
Source: PeerJ. 2020 Jan 3;8:e8280. doi: 10.7717/peerj.8280 (PMC6944124; doi:10.7717/peerj.8280)
Supplement: Supplemental Information 1 [file peerj-08-8280-s001.doc]

**Table S1**

| **sampling region** | **region identifier** | **N** | **no. *A.m. capensis* colonies sampled** | **no. *A.m. scutellata* colonies sampled** | **no. hybrid colonies sampled** |
| --- | --- | --- | --- | --- | --- |
| Beaufort West | BW | 30 (3) | 0 | 1 | 2* |
| Bloemfontein | BL | 32 (5) | 0 | 5 | 0 |
| Bredasdorp | BD | 29 (3) | 3 | 0 | 0 |
| Cape Town | CT | 30 (5) | 5 | 0 | 0 |
| Citrusdaal | CD | 29 (3) | 3 | 0 | 0 |
| East London | EL | 27 (3) | 2 | 1 | 0 |
| George | GE | 24 (3) | 3 | 0 | 0 |
| Graaff-Reinet | GR | 29 (3) | 3 | 0 | 0** |
| Grahamstown | GT | 29 (4) | 3 | 0 | 1 |
| Klawer | KL | 30 (5) | 3 | 0 | 2 |
| Knysna | KN | 30 (4) | 4 | 0 | 0 |
| Kroonstad | KR | 28 (4) | 0 | 4 | 0 |
| Laingsburg | LB | 26 (4) | 4 | 0 | 0 |
| Langebaan | LA | 30 (3) | 3 | 0 | 0 |
| Moorreesburg | MB | 30 (4) | 4 | 0 | 0 |
| Modderfontein | MF | 30 (3) | 3 | 0 | 0 |
| Oudtshoorn | OD | 30 (6) | 5 | 0 | 1 |
| Plettenburg Bay | PB | 30 (4) | 4 | 0 | 0 |
| Port Elizabeth | PE | 10 (1) | 1 | 0 | 0 |
| Pretoria | PT | 27 (3) | 0 | 3 | 0 |
| Riversdale | RD | 30 (3) | 3 | 0 | 0 |
| Saint Francis | SF | 20 (2) | 2 | 0 | 0 |
| Springbok | SP | 30 (3) | 0 | 3 | 0 |
| Stellenbosch | ST | 30 (3) | 3 | 0 | 0 |
| Swellendam | SW | 29 (4) | 4 | 0 | 0 |
| Touwsrivier | TR | 27 (3) | 3 | 0 | 0 |
| Upington | UP | 29 (4) | 0 | 4 | 0 |
| Vryburg | VR | 30 (3) | 0 | 3 | 0 |
| Worcester | WD | 28 (3) | 2 | 0 | 1 |
| *These hybrid colonies were more *A.m. scutellata*-like (i.e. had high *A.m. scutellata* scores despite their hybrid designation).  **Unsampled hybrid colonies were present in the region. | | | | | |
