# Supplementary material for: Genetic diversity and population structure of two subspecies of western honey bees (Apis mellifera L.) in the Republic of South Africa as revealed by microsatellite genotyping
Source: PeerJ. 2020 Jan 3;8:e8280. doi: 10.7717/peerj.8280 (PMC6944124; doi:10.7717/peerj.8280)
Supplement: Supplemental Information 2 [file peerj-08-8280-s002.doc]

**Table S2**

| multiplex groups | original marker name | primer name | nucleotide sequence  (5'–3') | repeat motive | fluorochrome | range of allele size (bp) | *N* *_Allele_* |
| --- | --- | --- | --- | --- | --- | --- | --- |
| **Plex 1** | A107 | A107-F  A107-R | 5'-CCGTGGGAGGTTTATTGTCG-3'  5'-GGTTCGaTAACGGATGACACC-3' | (CT)_23_ | FAM | 138 – 184 | 19.73 |
|  | A028 | A28-F  A28-R | 5'-GAAGAGCGTTGGTTGCAGG-3'  5'-GCCGTTCATGGTTACCACG-3' | (AG)_6_(GAG)_6_ | VIC | 128 – 134 | 11.4 |
|  | UN351 | UN351-F  UN351-R | 5'-AGCATACTTCTTCACCGAACC-3'  5'-TCCGTTTATGCTTCATTTTCGA-3' | (AT)_13_ | NED | 142 – 176 | 15.93 |
|  | AP081 | AP81-F  AP81-R | 5'-GGATCGTCGAGGCGTTGA-3'  5'-GAAAAGTATTCCGCCGAGCA-3' | (GT)_8_ | PET | 124 – 136 | 11.4 |
| **Plex 2** | A079 | A79-F  A79-R | 5'-CGAAGGTTGCGGAGTCCTC-3'  5'-GTCGTCGGACCGATGCG-3' | (CCT)_10_(GA)_10_ | FAM | 93 – 127 | 12.4 |
|  | A024 | A24-F  A24-R | 5'-CACAAGTTCCAACAATGC-3'  5'-CACATTGAGGATGAGCG-3' | (CT)_10_ | VIC | 92 – 106 | 6.6 |
|  | AP043 | AP43-F  AP43-R | 5'-GGCGTGCACAGCTTATTCC-3'  5'-CGAAGGTGGTTTCAGGCC-3' | (TA)_6_GATA(GA)_10_ | PET | 129 – 183 | 11.8 |
|  | HB-THE-03 | HB-THE-3-F  HB-THR-3-R | 5'-TAACTGGTCGTCGGTGTT-3'  5'-CACGTAGAGAATCCCATTGT-3' | (TA)_11_(TC)_12_ | NED | 174 – 202 | 11.57 |
| **Plex 3** | HB-THE-02 | HB-THE-2-F  HB-THE-2-R | 5'-GGGAAAGATATTAGGGAGGA-3'  5'-CGACGAAAAATTACAAGGAC-3' | (TA)_12_ | FAM | 236 – 256 | 11.73 |
|  | IM | IM-F  IM-R | 5'-ACGCAAATGACAAGTATTAG-3'  5'-GAGTGTATTTCGAAATCGATG-3' | (GA)_15_ | VIC | 174 – 202 | 11.3 |
|  | A014 | A14-F  A14-R | 5'-GTGTCGCAATCGACGTAACC-3'  5'-GTCGATTACCGATCGTGACG-3' | (CT)_13_(GGT)_9_ | PET | 209 – 262 | 14.03 |
| **Plex 4** | HB-C016-5 | HB-C16-5-F  HB-C16-5-R | 5'-ATTTTATGCGCGTTTCGTA-3'  5'-CATGGCTCCTCCATTAAATC-3' | (TC)_23_ | FAM | 65 – 89 | 8.43 |
|  | HB-SEX-01 | HB-SEX-1-F  HB-SEX-1-R | 5'-AGTGCAAAATCCAAATCATC-3'  5'-ATTCGATCACCCAAAGAA-3' | (A)_15_ | VIC | 142 – 165 | 9.73 |
|  | A088 | A88-F  A88-R | 5'-CGAATTAACCGATTTGTCG-3'  5'-GATCGCAATTATTGAAGGAG-3' | (CT)_10_(GGA)_7_ | NED | 136 – 149 | 11.9 |
|  | A007 | A7-F  A7-R | 5'-GTTAGTGCCCTCCTCTTGC-3'  5'-CCCTTCCTCTTTCATCTTCC-3' | (CT)_3_(T)_7_(CT)_24_ | PET | 95 – 117 | 12.8 |
| **Plex 5** | AP066 | AP66-F  AP66-R | 5'-TTGCATTCGGTCTCCAGC-3'  5'-ACTTGCCGCGGTATCTGA-3' | (CT)_11_ | FAM | 90 – 102 | 10.9 |
|  | AC006 | AC6-F  AC6-R | 5'-GATCGTGGAAACCGCGAC-3'  5'-CACGGCCTCGTAACGGTC-3' | (TCT)_5_(TTC)_10_ | VIC | 144 – 166 | 12.66 |
|  | B124 | B124-F  B124-R | 5'-GCAACAGGTCGGGTTAGAG-3'  5'-CAGGATAGGGTAGGTAAGCAG-3' | (CT)_8_(CT)_14_(GGCT)_8_ | NED | 212 – 262 | 13.56 |
| **Plex 6** | HB-THE-04 | HB-THE-4-F  HB-THE-4-R | 5'-GCTGGAAGGGAACTGTAGA-3'  5'-GGACGCGTTTTAATATCTCA-3' | (GA)_9_ | NED | 225 – 239 | 7.93 |
