# Supplementary material for: Genetic diversity and population structure of two subspecies of western honey bees (Apis mellifera L.) in the Republic of South Africa as revealed by microsatellite genotyping
Source: PeerJ. 2020 Jan 3;8:e8280. doi: 10.7717/peerj.8280 (PMC6944124; doi:10.7717/peerj.8280)
Supplement: Supplemental Information 3 [file peerj-08-8280-s003.doc]

**Table S3**

| **BL** | **KR** | **PT** | **SP** | **UP** | **VR** | **BD** | **CT** | **GE** | **LB** | **LA** | **MB** | **OD** | **RD** | **ST** | **SW** | **TR** | **EL** | **GR** | **GT** | **KL** | **KN** | **MF** | **PB** | **SF** |  |
| --- | --- | --- | --- | --- | --- | --- | --- | --- | --- | --- | --- | --- | --- | --- | --- | --- | --- | --- | --- | --- | --- | --- | --- | --- | --- |
| 0.00 |  |  |  |  |  |  |  |  |  |  |  |  |  |  |  |  |  |  |  |  |  |  |  |  | **BL** |
| 0.40 | 0.00 |  |  |  |  |  |  |  |  |  |  |  |  |  |  |  |  |  |  |  |  |  |  |  | **KR** |
| 0.37 | 0.44 | 0.00 |  |  |  |  |  |  |  |  |  |  |  |  |  |  |  |  |  |  |  |  |  |  | **PT** |
| 0.38 | 0.45 | 0.42 | 0.00 |  |  |  |  |  |  |  |  |  |  |  |  |  |  |  |  |  |  |  |  |  | **SP** |
| 0.39 | 0.54 | 0.54 | 0.37 | 0.00 |  |  |  |  |  |  |  |  |  |  |  |  |  |  |  |  |  |  |  |  | **UP** |
| 0.34 | 0.44 | 0.43 | 0.49 | 0.48 | 0.00 |  |  |  |  |  |  |  |  |  |  |  |  |  |  |  |  |  |  |  | **VR** |
| 0.53 | 0.46 | 0.52 | 0.50 | 0.63 | 0.57 | 0.00 |  |  |  |  |  |  |  |  |  |  |  |  |  |  |  |  |  |  | **BD** |
| 0.49 | 0.48 | 0.46 | 0.50 | 0.55 | 0.54 | 0.57 | 0.00 |  |  |  |  |  |  |  |  |  |  |  |  |  |  |  |  |  | **CT** |
| 0.54 | 0.48 | 0.46 | 0.53 | 0.55 | 0.62 | 0.54 | 0.49 | 0.00 |  |  |  |  |  |  |  |  |  |  |  |  |  |  |  |  | **GE** |
| 0.47 | 0.40 | 0.42 | 0.40 | 0.44 | 0.49 | 0.37 | 0.41 | 0.41 | 0.00 |  |  |  |  |  |  |  |  |  |  |  |  |  |  |  | **LB** |
| 0.42 | 0.45 | 0.50 | 0.43 | 0.47 | 0.44 | 0.53 | 0.45 | 0.48 | 0.36 | 0.00 |  |  |  |  |  |  |  |  |  |  |  |  |  |  | **LA** |
| 0.48 | 0.55 | 0.38 | 0.48 | 0.53 | 0.56 | 0.51 | 0.47 | 0.46 | 0.39 | 0.47 | 0.00 |  |  |  |  |  |  |  |  |  |  |  |  |  | **MB** |
| 0.48 | 0.55 | 0.46 | 0.45 | 0.51 | 0.49 | 0.42 | 0.49 | 0.40 | 0.39 | 0.41 | 0.45 | 0.00 |  |  |  |  |  |  |  |  |  |  |  |  | **OD** |
| 0.48 | 0.47 | 0.43 | 0.46 | 0.68 | 0.55 | 0.30 | 0.52 | 0.47 | 0.39 | 0.47 | 0.35 | 0.39 | 0.00 |  |  |  |  |  |  |  |  |  |  |  | **RD** |
| 0.56 | 0.50 | 0.51 | 0.56 | 0.63 | 0.57 | 0.44 | 0.50 | 0.44 | 0.44 | 0.42 | 0.52 | 0.49 | 0.38 | 0.00 |  |  |  |  |  |  |  |  |  |  | **ST** |
| 0.39 | 0.45 | 0.44 | 0.44 | 0.56 | 0.49 | 0.49 | 0.45 | 0.49 | 0.39 | 0.50 | 0.46 | 0.46 | 0.44 | 0.54 | 0.00 |  |  |  |  |  |  |  |  |  | **SW** |
| 0.50 | 0.58 | 0.51 | 0.46 | 0.57 | 0.65 | 0.55 | 0.62 | 0.46 | 0.46 | 0.45 | 0.36 | 0.34 | 0.45 | 0.48 | 0.43 | 0.00 |  |  |  |  |  |  |  |  | **TR** |
| 0.47 | 0.38 | 0.54 | 0.56 | 0.63 | 0.43 | 0.49 | 0.61 | 0.47 | 0.39 | 0.47 | 0.57 | 0.46 | 0.46 | 0.57 | 0.52 | 0.58 | 0.00 |  |  |  |  |  |  |  | **EL** |
| 0.44 | 0.48 | 0.50 | 0.50 | 0.57 | 0.48 | 0.42 | 0.55 | 0.44 | 0.40 | 0.54 | 0.56 | 0.43 | 0.44 | 0.54 | 0.47 | 0.60 | 0.43 | 0.00 |  |  |  |  |  |  | **GR** |
| 0.42 | 0.45 | 0.39 | 0.35 | 0.42 | 0.49 | 0.46 | 0.44 | 0.41 | 0.38 | 0.38 | 0.39 | 0.47 | 0.48 | 0.53 | 0.41 | 0.41 | 0.47 | 0.45 | 0.00 |  |  |  |  |  | **GT** |
| 0.34 | 0.41 | 0.40 | 0.38 | 0.36 | 0.47 | 0.42 | 0.40 | 0.45 | 0.34 | 0.40 | 0.39 | 0.39 | 0.45 | 0.42 | 0.38 | 0.40 | 0.48 | 0.46 | 0.34 | 0.00 |  |  |  |  | **KL** |
| 0.36 | 0.52 | 0.34 | 0.47 | 0.53 | 0.40 | 0.42 | 0.48 | 0.39 | 0.35 | 0.32 | 0.35 | 0.32 | 0.43 | 0.46 | 0.40 | 0.41 | 0.43 | 0.43 | 0.34 | 0.40 | 0.00 |  |  |  | **KN** |
| 0.46 | 0.47 | 0.50 | 0.38 | 0.56 | 0.51 | 0.50 | 0.54 | 0.58 | 0.47 | 0.55 | 0.45 | 0.59 | 0.56 | 0.68 | 0.53 | 0.57 | 0.53 | 0.45 | 0.43 | 0.45 | 0.48 | 0.00 |  |  | **MF** |
| 0.41 | 0.45 | 0.41 | 0.48 | 0.52 | 0.42 | 0.49 | 0.45 | 0.40 | 0.44 | 0.43 | 0.48 | 0.42 | 0.42 | 0.43 | 0.41 | 0.53 | 0.40 | 0.41 | 0.37 | 0.40 | 0.37 | 0.45 | 0.00 |  | **PB** |
| 0.40 | 0.47 | 0.46 | 0.52 | 0.59 | 0.46 | 0.41 | 0.52 | 0.47 | 0.47 | 0.43 | 0.58 | 0.48 | 0.40 | 0.49 | 0.41 | 0.51 | 0.40 | 0.40 | 0.36 | 0.43 | 0.37 | 0.47 | 0.37 | 0.00 | **SF** |
